# Valorisation of Pineapple Cannery Waste as a Cost Effective Carbon Source for Poly 3-hydroxyabutyrate (P3HB) Production

**DOI:** 10.3390/polym15153297

**Published:** 2023-08-04

**Authors:** Waranya Suwannasing, Varavut Tanamool, Pakjirat Singhaboot, Pakawadee Kaewkannetra

**Affiliations:** 1 Department of Intellectual Property, Ministry of Commerce, Nonthaburi 11000, Thailand; waranya.s@ipthailand.go.th; 2 Chemistry Program, Faculty of Science and Technology, Nakhon Ratchasima Rajabhat University, Nakhon Ratchasima 30000, Thailand; varavut.t@nrru.ac.th; 3 Faculty of Agricultural Product Innovation and Technology, Srinakharinwirot University, Nakhon Nayok 26120, Thailand; pakjirat@g.swu.ac.th; 4 Department of Biotechnology, Faculty of Technology, Khon Kaen Univerisity, Khon Kaen 40002, Thailand

**Keywords:** pineapple processing waste, polymer, polyhydroxyalkanoates (PHAs), fermentation

## Abstract

Pineapple is one of the most important agro-industrial sugar-based fruits in Thailand. In this study, the waste stream from pineapple cannery processing was utilised and evaluated for potential use in the production of a main biopolymer group widely known as polyhydroxyalkanoates (PHAs) through aerobic batch fermentation. Firstly, pineapple cannery waste (PCW) collected from three processing sources, pineapple juice (PAJ), peel and core juice (PCJ), and pulp-washing water (PWW), was used as a carbon source. Secondly, it was characterised and pretreated. Then, batch fermentation was performed by using the optimal condition (200 rpm agitation rate, 37 °C, and fermentation time of 72 h) under two different nutrient conditions in each type of carbon source. The results revealed that PHAs were produced during 24–72 h of fermentation without any interference. The PHAs product obtained was characterised by their properties. Interestingly, GC-MS showed homopolymer of poly 3-hydroxybutyrate (P3HB) group characteristics, such as OH, CH, and C=O; meanwhile, H^1^ NMR analysis showed signals corresponding to CH_3_, CH_2_, and CH, respectively. Remarkably, utilising the PCW showed a high-potential cheap carbon source for the production of PHAs as well as for the treatment of wastewater from the fruit industry.

## 1. Introduction

**Figure 1:**
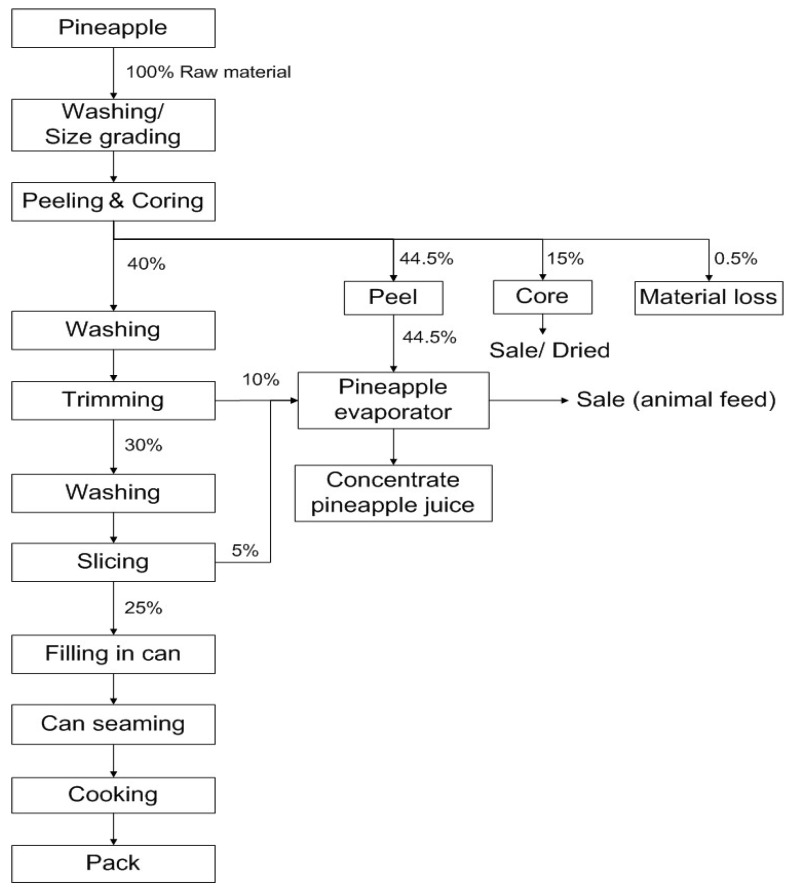
The schematic diagram of pineapple cannery process.

Polyhydroxyalkanoates (PHAs), one of the main biopolymer groups, are a family of diverse linear polyesters. Naturally, PHAs are produced and accumulated in the cells by different species of bacteria, archaea, and plants [1]. However, more than 150 bacterial strains can utilise renewable sugar-based materials and accumulate PHAs granules in their cells as energy sources when they undergo stress conditions of growth that are in excess of carbon sources and limited nutrients, i.e., nitrogen, phosphorus, etc. The advantageous properties of PHA polymer are that it is biodegradable, biocompatible, and compostable. The fact that petroleum-based plastics are non-biodegradable materials increases the demand for petroleum, and hence their price. Therefore, PHAs are considered as alternative and environmentally friendly materials that are similar to the synthetic plastic polypropylene (PP) [2]. They are used to replace non-degradable plastics to reduce environmental problems and improve solid waste management. Although they have been developed rapidly in various applications such as biodegradable plastics for packaging materials, implant biomaterials, fibers, drug delivery carriers, etc., the major obstacle for their application, however, is the cost of production. This barrier causes PHAs to be priced uncompetitively and higher than petroleum-based polymers. In order to reach an economical price, improvement in the effective cost of PHAs has been extensively investigated both in upstream and downstream processes. In particular, the cost of raw materials during the upstream processes of PHAs production is about 50% of the total production cost. Typically, pure types of sugar such as glucose, fructose, and even sucrose are suitable to be used as carbon sources for PHAs production by various bacteria. However, the prices of sugars compared to other materials are still expensive. It is possible for the production of PHAs to compete with that of conventional plastics if they can be produced from inexpensive raw materials [3]. Economical raw materials that have been used and investigated for PHA production include cheap renewable industrial byproducts and even wastes [2,3]. According, pineapple (*Ananus comosus*) is one of the most important fruits in Thailand. It is mainly manufactured on a commercial scale in the form of canned fruits, juices, concentrates, jams, and frozen products [4,5]. Pineapple waste, a sugar-rich and sustainable material, has been used for the manufacturing of other products to increase the price of new products and to decrease the volume of waste. Figure 1, showing a schematic diagram of the pineapple cannery process modified from the Department of Industrial Works (DIW) of Thailand, illustrates that waste and byproducts are generated from raw materials at a rate of about 60% [6]. Information on exported pineapple products reported that pineapple cannery products from Thailand were exported at an average of 2 million tons/year (collected data from 2019 to 2020). Thus, as much as 1 million tons of waste is generated yearly [7]. 

Typically, pineapple waste can be converted into a variety of value-added products such as enzymes, fertiliser, animal feed, etc. The different parts of pineapple waste have different properties, for example, peel waste, a rich source of cellulose, hemicelluloses, and other carbohydrates, was employed to produce paper, cloth, and banknotes. Furthermore, core waste could be converted into alcoholic beverages or vinegar [8]. In addition, it has been reported that pineapple waste could be used as a substrate for the production of cellulose, methane, ethanol, citric acid, antioxidant compounds, and bioactive compounds, including a biopolymer of PHAs [9,10,11,12,13,14,15,16,17]. Recently, alternative means of pineapple waste utilisation were its uses as sources of proteolytic enzymes in which the different parts of pineapple would present different bromelain and other cysteine protease enzymes [18,19]. 

Nevertheless, there is no literature review about the utilisation of pineapple waste from pineapple cannery process to convert into a value added PHAs biopolymer product. Therefore, in this study, PHAs production was focused on the feasibility of pineapple waste to be used as a sole carbon source with/without nutrients adding conditions. Not only was the effect of nutrients investigated, but also the effect of different waste sources was determined. Since, pineapple waste occurred from different processes, the composition of the waste was different. Thus, the production of PHAs biopolymer via aerobic batch fermentation using an isolated Gram-positive strain of *Bacillus* sp. SV13 was performed. This strain showed a good and desirable character for producing biopolymer for medical and pharmaceutical applications. Differing from Gram-negative, the Gram-positive bacteria could produce the PHAs biopolymer with a lack of the lipopolysaccharides (LPS) that can induce a strong immunogenic reaction [20]. In order to determine the best condition for PHAs polymer production, a statistical test was applied to assist for data analysis using a twoway ANOVA test. In addition, the conversion efficiency of pineapple cannery waste into PHAs product was evaluated in terms of PHAs concentration, content, yield, and productivity. 

## 2. Materials and Methods

### 2.1. PHAs-Producing Bacterial Strain

A pure strain of *Bacillus* sp. SV13, isolated from the soil in sugarcane plantation area of Khon Kaen province, northeastern Thailand, was used as a PHAs-producing strain throughout this study [21]. The strain can employ various types of sugar, grow in a short-lag phase, and reach the stationary phase within 24 h when a nutrient medium is used. To prepare an inoculum, a loop of a single colony was inoculated into an Erlenmayer flask containing nutrient medium. It was incubated in a rotary incubator shaker (VS-8480 SFN, Bucheon-si, Gyeonggi-do, Republic of Korea) at 200 rpm agitation rate, at 37 °C for 10–12 h. Then, 10% (*v*/*v*) was used as a seed starter for PHAs production via aerobic batch fermentation.

### 2.2. Characterisation and Preparation of Pineapple Waste

In order to use pineapple cannery waste (PCW) as a sole carbon source, the PCW was collected and categorised into 3 types: pineapple juice (PAJ), peel and core (PCJ), and pulp-washing water (PWW). The PAJ was generated by trimming and slicing processes; meanwhile, the PCJ was generated by peeling and coring processes. Both of them were made in juice form by cutting and crushing and the solid was separated by filtration. Another PWW was generated from the slicing, trimming, and cutting processes. To control the PWW properties throughout the study, it was prepared by soaking method. In brief, 1 kg pulp was soaked in 10 L water for 1 h. Using this method, water quality of WPP would be similar in quality to the wastewater generated from pineapple cannery process which was estimated and reported by DIW (2021) [6]. Each type of waste was analysed and characterised for acidity-alkalinity, total sugar, sugar content, and also parameters indicating wastewater quality such as total soluble solid, total nitrogen (TN), total phosphorus (TP), suspended solid (SS), and biochemical oxygen demand (BOD) following the standard methods set by APHA, AWWA (2017) [22]. All wastes were kept in −20 °C prior to use as carbon source.

### 2.3. Fermentation of Pineapple Cannery Waste (PCW)

To study the production of PHAs by various carbon sources, frozen PAJ, PCJ, and PWW were thawed at room temperature before adjusting an initial pH to 6.0–6.5. Two conditions of nutrients and no nutrients addition were investigated in each type of carbon source. The nutrients used in this study consisted of 4 main components, (NH_4_)_2_SO_4_, and KH_2_PO_4_ for 1.5 g L^−1^ in each, Na_2_HPO_4_ 1.8 g L^−1^, together with the addition of 1 mL trace elements solution (TES). It should be noted that 1 L TES was prepared from 0.18 g ZnSO_4_∙7H_2_O, 0.37 g FeSO_4_∙7H_2_O, 0.55 g CoCl_2_∙6H_2_O, 0.30 g MnSO_4_∙5H_2_O, 0.16 g CuSO_4_∙5H_2_O, and 0.12 g Na_2_MoO_4_∙2H_2_O. Then, 10 mL inoculum was transferred into the 250 mL Erlenmeyer flask containing 90 mL with different sole carbon sources to be used as PHAs production medium. Then, they were fermented under the following conditions: 200 rpm of agitation rate, at 37 °C, and for 72 h. The samples were withdrawn every 12 h to analyse the PHAs concentration, dry cell mass (DCM), total sugar, and sugar content [21]. 

### 2.4. PHAs Production in Fermentor

To scale up the production of PHAs, a 5 L fermentor (Sartorious Biostat B, Gottingen, Germany) was performed with 3.5 L working volume. The fermentation condition was controlled as follows: 37 °C, 250 rpm agitation rate, pH 6.25, and 1.5 vvm aeration rate. The samples were withdrawn for 24 h. Then, PHAs concentration, DCM, and total sugar were determined. 

### 2.5. Analytical Methods

#### 2.5.1. Dry Cell Mass (DCM) Measurement

The fermented broth was withdrawn and was then centrifuged using 10,000× *g* at 20 °C for 10 min (Changsha TGL-16 C, Changsha, China). The biomass was estimated in the form of dried cells by gravimetric method [23]. 

#### 2.5.2. Determination of Sugar Concentration and Sugar Consumption

Total sugar was analysed by using the phenol-sulphuric method [24]. The sugar type was analysed using High Performance Liquid Chromatography (HPLC) (Verti Sep^TM^ GES, NH2 column; RID detector, Shimazu, Kyoto, Japan) with chromatographic conditions of 40 °C; 90:10 of acetonitrile; water; and 0.75 mL/min flow of mobile phase. 

#### 2.5.3. Water Quality Analysis

Water quality was analysed following the standard methods for the examination of water and wastewater [22]. Parameters of total nitrogen (TN) and total phosphorus (TP) were analysed by total kjeldahl nitrogen (TKN) and stannous chloride methods. Suspended solid (SS) was determined at 105 °C for 1 h using glass fiber filter. Meanwhile, a parameter of BOD was analysed by azide modification method at 20 °C for 5 days compared to 0 day. 

#### 2.5.4. PHAs Recovery

The modified gravimetrical method described by Chala et al. (2019) [25] was used to determine the extracted PHAs. The sequence steps were explained as follows: wet cell mass or cell pellet obtained from centrifugation was treated with 3% (*v*/*v*) sodium hypochlorite (commercial grade bleach), incubated at 37 °C and stirred for 1 h. Thereafter, chloroform was added into mixed solution and was then stirred at 50 + 5 °C for 3 h. An upper phase of chloroform was separated and evaporated. Then, white powder/sheet was left at room temperature until it reached a constant weight. The extracted PHAs were measured by weighing.

#### 2.5.5. PHAs Characterisation and Properties Analysis

Chemical structures of recovered PHAs polymer were characterised by using Nuclear Magnetic Resonance (NMR) technique. The PHAs samples were dissolved in chloroform-d (CDCl_3_) and were then analysed by 400 MHz NMR Spectrometer (Varian Bruker, NMR-400 MHz, Billerica, MA, USA) at 25 °C. The chemical shifts of ^13^C-NMR were reported in parts per million (ppm). It should be noted that, PHAs are mostly found in the form of poly (3-hydroxybuty rate) or P3HB. A standard analytical grade of P3HB was analysed and compared to the PHAs samples. The thermal properties of melting temperature (T_m_) and enthalpy of fusion (∆H) were measured using the differential scanning calorimeter (DSC) (DSC 823e, Mettler Toledo, Greifensee, Switzerland). The samples were operated at a temperature scan of 10 °C/min from −20 to 200 °C. The values of T_m_ and ∆H were obtained from scanned DSC thermograms. In addition, the crystallinity of PHAs was determined by the ∆H of 100% crystallinity of P3HB which was assumed to be 146 J/g [26].

### 2.6. Statistical Analysis

Statistical analysis was performed by using Minitab 16 software (Trial version) to evaluate the effects of nutrient adding and different waste sources on the PHAs production. The data were analysed in terms of PHAs concentration, PHAs content (Y_p/x_), PHAs yield (Y_p/s_), and productivity. An analysis of variance (ANOVA) was used to test the means of the different treatments and the hypothesis (α = 0.05).

## 3. Results and Discussion

### 3.1. Pineapple Waste Characterisation

The compositions of pineapple waste generated from the different steps of the pineapple cannery process presented an acidic property for all waste sources. PAJ, PCJ, and PWW consisted of three types of sugar including fructose, glucose, and sucrose. The total sugar found in PAJ was similar to the sugar content of PCJ, while PWW showed the lowest. Other parameters (pH, BOD, TKN, SS, and total soluble solid) presented similar values for PAJ and PCJ, while PWW showed the lowest values for all parameters, excluding pH value (see Table 1).

The results revealed that the components of waste, i.e., sugar concentration, nitrogen, and phosphorus, were possible for use as a medium for PHAs production, since the sugar content in the culture media was typically prepared at about 10–20 g L^−1^ for employment as a carbon source for bacterial growth. Furthermore, other nutrients (ascorbic acid, manganese, magnesium, copper, iron, potassium, etc.) contained in pineapple were profitable and possibly play an important role as growth factors for bacterial strains. It should be noted that the condition used for PHAs accumulation were an excess in carbon source and a limiting of nutrients supplements, such as nitrogen and phosphorus, that were shown in terms of TKN and TP.

### 3.2. PHAs Production in a Shake Flask

**Figure 2:**
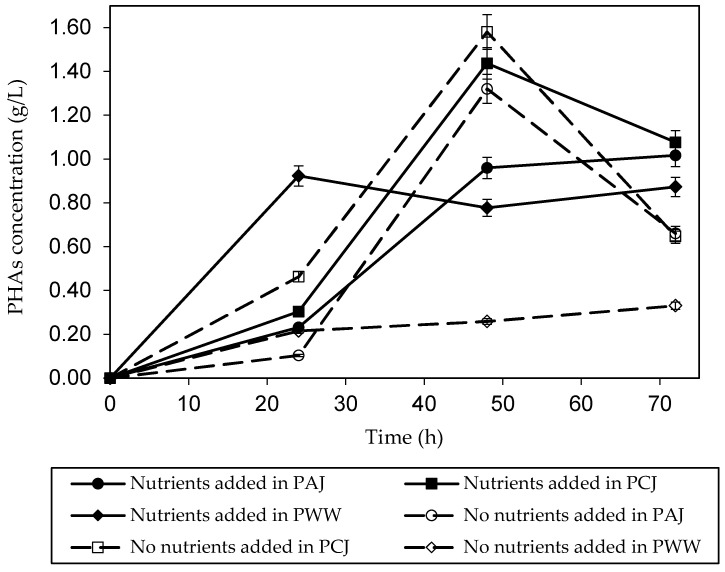
The profile of PHAs production from pineapple waste by *Bacillus* sp. SV13 via aerobic batch fermentation with/without adding nutrients.

The PAJ and PCJ trends were shown to have similar PHAs concentrations both when nutrients/no nutrients were added. PHAs concentration reached the highest value at 48 h and then the production slowly declined. Unlike in PWW with nutrients added, the production of PHAs increased gradually in PHAs concentration for the first 24 h and then the production showed a steady concentration of PHAs. It should be noted that the experiments were carried out in triplication for reproducibility. The data collected were taken and shown as mean and error bar patterns (see Figure 2).

For the effect of high sugar concentration, PAJ and PCJ containing a high concentration of sugar may provide unsuitable carbon sources for bacterial growth. Thus, both of them showed a slower growth than in the case of PWW. During fermentation, acidity and alkalinity properties showed that the pH dropped to 5.0 + 0.5 in all conditions. It decreased dramatically during the first 24 h. Furthermore, the fermented broth after 72 h was considered in terms of TKN and TP. It was revealed that TKN and TP decreased in all sources of pineapple waste (data not shown). In particular, using PWW as a sole carbon source without adding nutrients could reduce nitrogen from 420 to 105 mg/L. Meanwhile, TP showed a slight decrease in concentration. This value was close to the industrial effluent standard (100 mg/L). A previous study mentioned the effect of using inorganic and organic nitrogen sources. It was indicated that the utilisation of nitrogen by *B. megaterium* in an inorganic form (ammonium chloride) would be better than an organic form. This was in agreement with this study; TKN and TP were still detected and they were not completely utilised [27].

### 3.3. Statistical Test of PHAs Production from Different Waste Sources

The results obtained from all conditions are summarised and shown in Table 2. Each parameter was reported in terms of DCM, PHAs concentration, Y_p/s_,% PHAs content, and volumetric productivity. The PAJ and PCJ showed high values for almost all parameters. The PWW without nutrients reached low values of DCW, PHAs concentration, and productivity. On the other hand, the PWW with nutrients showed the highest values for all parameters.

The results were compared to a previous study of PHAs production by *Bacillus* strains from sugar-based carbon sources. It showed that PHAs content reached about 16–61% [28]. Similarly, this study revealed that pineapple waste gave a PHAs content of about 20–60%. Interestingly, PHAs production by *Bacillus* sp. SV13 using a low concentration of total sugar (PWW) gave the maximum PHAs content of 65.95%, which was higher than PHAs production (35% PHAs content) by *B. cereus* YB-4 using LB medium plus glucose [29]. Furthermore, a comparison of PHAs production using agricultural materials and wastes is summarised in Table 3. It was found that pineapple waste showed a possibility for use as a carbon source.

### 3.4. PHAs Production in a Fermentor

**Figure 3:**
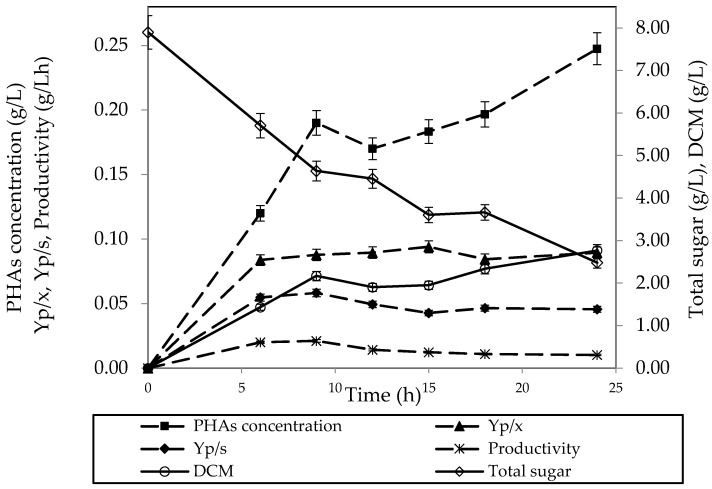
The profile of PHAs production via a batch fermentation in a 5 L fermentor by using PWW with added nutrients.

In Figure 3, the PHAs concentration showed no significant differences among waste sources used. Thus, only an aerobic batch fermeation for PHAs production was performed using PWW as a carbon source. The profile of PHAs production over 24 h of fermentation showed a sharp increasing trend in the case of PHAs concentration for the first 9 h. Then, all trends of all parameters showed little fluctuation, except for the case of total sugar, as sugar concentration decreased continuously from 8 g/L to 2 g/L. After 24 h of fermentation, the maximum PHAs concentration was obtained at about 0.25 g/L and a higher PHAs concentration could be achieved due to the upward trend of the PHAs production. It should be noted that all trends, obtained from the experimental data in triplication, were means and standard deviation of error.

### 3.5. Characterisation of PHAs

**Figure 4:**
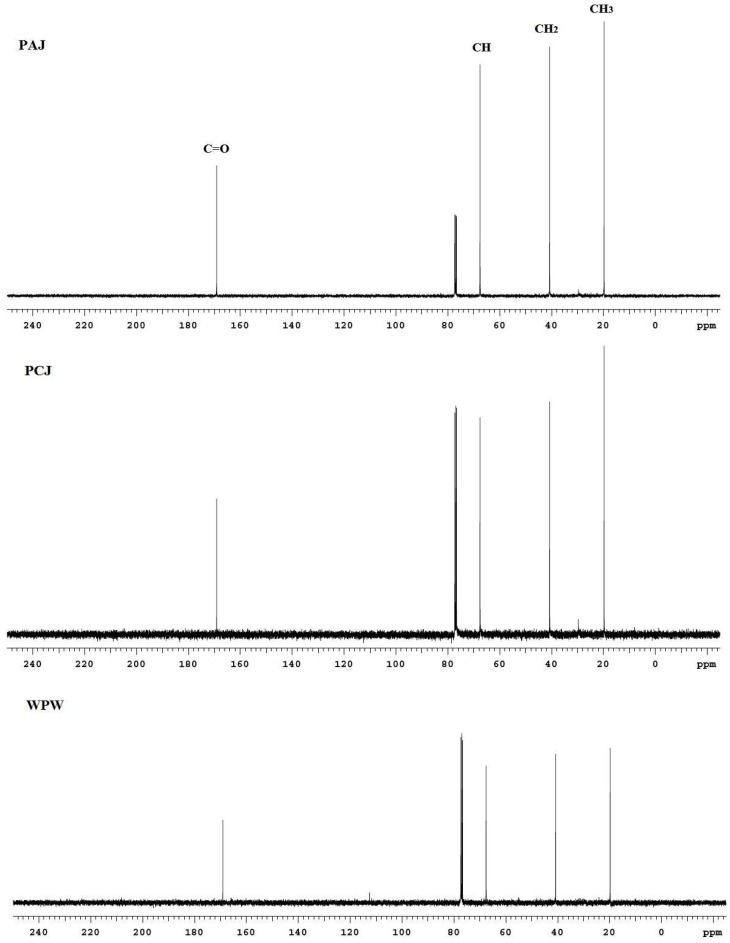
The ^13^C-NMR spectra of PHAs polymer extracted from PAJ, PCJ, and PWW with added nutrients.

The chemical structure of PHAs polymers was identified using ^13^C-NMR and ^1^H-NMR techniques. The PHAs product obtained from various sources of pineapple wastes were proven to be a homopolymer of polyhydroxybutyrate (PHB). It should be noted that PHB was in the form of poly-3-hydroxybutyrate (P3HB). The^13^C-NMR spectra for all PHAs samples presented in spectra similar to an analytical grade of poly[(R)-3-hydroxybutyric acid] standard. The chemical shifts were presented as follows: CH_3_ at 19.74 ppm, CH_2_ at 40.77 ppm, CH at 67.59 ppm, and C=O at 69.11 ppm. The ^1^H-NMR spectra of all PHAs samples was confirmed to be similar to the PHB standard. Regarding the results of the NMR spectra, both ^13^C-NMR and ^1^H-NMR were confirmed to have the same patterns to the PHB standard spectra. The results obtained were in agreement with previous works [15,16,17]. The example of NMR spectra and ^13^C-NMR spectra of PHAs, in the case of nutrients addition, is shown in Figure 4. 

In cases of PHAs productivity and yield, the maximum for each term was found at 0.021 gL^−1^·h^−1^ and 0.058 g/g after 9 h of fermentation. Considering the DCM, it was greater than in case of a flask scale. It was implied that the bacterium could use sugar and nutrients for growth better than PHAs accumulation. Additionally, under the limitation of nutrients and stress conditions that were suitable to induce the production of PHAs. Therefore, a mild condition of pH of 6.25 during the fermentation could induce cell mass production rather than PHAs production, although PHAs concentration in a fermentor was less than a flask scale. However, there was no difference in terms of productivity and capacity to utilise sugar. This might change in environmental condition during fermentation such as pH, agitation type, aeration rate, etc.

Besides the PHAs structure, the *T*_m_, ∆*H*, and crystallinity of PHAs polymer from pineapple waste were analysed and summarised in Table 4. The *T*_m_ value of all samples gave a slight difference from pure PHB (176.41 °C) which corresponded to the melting point of 177 °C reported by Tri et al. (2013) [35]. The ∆*H* was found in the range of 60 to 80 J/g, while the pure PHB showed at about 94.57 J/g. The result of crystallinity was demonstrated as being in the range of 42 to 55%. The highest values of *T*_m_, ∆*H*, and crystallinity were found in the PHAs polymer produced from PWW with nutrient addition. The results revealed that the properties of recovered PHAs polymer from pineapple waste showed little difference. It is possible that the bacterial growth conditions and product recovery steps (i.e. cell disruption and extraction) might be affected on these properties. The results obtained could be useful for further applications. For example, different *T*_m_ implied a maximum temperature and the point that PHAs were changed from the soild into the liquid phase. While other properties, such as a low crystallinity, produced less stiff and brittle properties.

## 4. Conclusions

Pineapple waste successfully and obviously showed potential for use as a sole carbon source for producing a homo-biopolymer of PHAs. Furthermore, it was also feasible to reduce the organic loading rate in wastewater. Moreover, it was considered a challenge as a sugar-based material for large scale PHAs production due to its price and quantity. However, process optimisation, using the response surface methodology (RSM), will be the focus of future work. 

## Figures and Tables

**Table 1:** Characterisation of pineapple waste.

Sources of Waste	pH	BOD * (mgL^−1^)	TKN ** (mgL^−1^)	TP *** (mgL^−1^)	SS **** (mgL^−1^)	Total Soluble Solid (°Brix )	Sugar Content (gL^−1^)			Total Sugar (gL^−1^)
							Fructose	Glucose	Sucrose	
PAJ	3.6	26,000	1120	45	16,762	14.8	20.30	18.88	78.45	117.62
PCJ	3.7	28,000	840	51	14,053	12.0	23.34	26.08	50.85	100.28
PWW	4.6	1700	420	6.8	627	0.8	1.64	1.38	7.24	10.26

* BOD (biochemical oxygen d0emand), ** TKN (total Kjeldahl nitrogen), *** TP (total phosphorus, **** SS (suspended solid).

**Table 2:** Comparison of different pineapple waste sources for PHAs production via fermentation (24–72 h) by *Bacillus* sp. SV13 under nutrient addition and no nutrient addition.

Nutrient Addition	Sources of Waste	DCM (g/L)	PHAs Concentration (g/L)	Y_p/s_ (g/g)	% PHAs Content (*w*/*w*)	Productivity (g/Lh)
Nutrient addition	PAJ	4.59 ± 2.66	0.74 ± 1.09	0.030 ± 0.03	17.81 ± 30.35	0.015 + 0.01
	PCJ	3.87 ± 1.21	0.94 ± 1.44	0.077 ± 0.12	23.69 ± 35.57	0.019 + 0.02
	PWW	1.57 ± 0.94	0.86 ± 0.18	0.216 ± 0.11	56.45 ± 28.63	0.022 + 0.04
No nutrient addition	PAJ	4.06 ± 1.81	0.69 ± 1.51	0.054 ± 0.34	19.45 ± 48.14	0.014 + 0.03
	PCJ	3.63 ± 1.44	0.90 ± 1.49	0.127 ± 0.28	24.48 ± 38.97	0.020 + 0.03
	PWW	0.78 ± 0.19	0.27 ± 0.14	0.047 ± 0.04	34.00 ± 10.71	0.006 + 0.01

**Table 3:** Comparison of the PHAs production (PHAs concentration and PHAs content) from various agricultural materials and wastes.

Carbon Sources	PHAs Concentration (g/L)	PHAs Content (%*w*/*w*)	Microorganisms	References
Cheese whey	1.50	51.00	*Bacillus megaterium* CCM 2037	Obruca, et al., 2011 [30]
Sugar beet juice	4.0 ± 0.95	38.66 ± 7.28	*Alcaligenes latus*	Wang, et al., 2013 [31]
Sugarcane juice	1.84	30.60	*Alcaligenes eutrophus*	Suwannasing, et al., 2015 [21]
Sugarcane molasse	0.67–30.52	42.10–61.60	*Bacillus megaterium* DSM 90	Vu, et al., 2021 [32]
Sweet sorghum juice	1.74	57.62	*Bacillus aryabhattai*	Tanamool, et al., 2013 [33]
Sugarcane molasses	2.25	43.00	*Bacillus licheniformis* DSM 394	Gojgic-Cvijovic, et al., 2019 [34]
Pineapple waste	1.50	65.95	*Bacillus* sp. SV13	This work

**Table 4:** The properties of PHAs polymer production from pineapple waste by *Bacillus* sp. SV13.

Nutrient Addition	Sources of Waste	Melting Temperature, *T*_m_ (°C)	Enthalpy of Fusion, ∆*H* (J/g)	Crystallinity ^a^ (%)
Nutrient addition	PAJ	170.0	67.08	45.94
	PCJ	171.4	61.67	42.24
	PWW	171.8	80.31	55.01
No nutrient addition	PAJ	168.15	77.15	52.84
	PCJ	168.51	79.00	54.11
	PWW	173.0	81.10	55.55

^a^ The crystallinity was calculated from the ratio of enthalpy of fusion of PHAs samples and the enthalpy of fusion of 100% crystallinity of PHB (146 J/g).

## Data Availability

Not applicable.
